# Objective Methods of Assessing Fluid Status to Optimize Volume Management in Kidney Disease and Hypertension: The Importance of Ultrasound

**DOI:** 10.3390/jcm12196368

**Published:** 2023-10-05

**Authors:** Sharad Patel, Adam Green, Sandhya Ashokumar, Andrew Hoke, Jean-Sebastien Rachoin

**Affiliations:** 1 Division of Critical Care Medicine, Cooper University Health Care, Camden, NJ 08103, USA; patel-sharad@cooperhealth.edu (S.P.); green-adam@cooperhealth.edu (A.G.); ashokumar-sandhya@cooperhealth.edu (S.A.); 2 Department of Medicine, Cooper Medical School of Rowan University, Camden, NJ 08103, USA; 3 Department of Medicine, Cooper University Health Care, Camden, NJ 08103, USA; hoke-andrew@cooperhealth.edu

**Keywords:** volume overload, fluid overload, ultrasound, echography, sonography

## Abstract

Fluid overload, a prevalent complication in patients with renal disease and hypertension, significantly impacts patient morbidity and mortality. The daily clinical challenges that clinicians face include how to identify fluid overload early enough in the course of the disease to prevent adverse outcomes and to guide and potentially reduce the intensity of the diuresis. Traditional methods for evaluating fluid status, such as pitting edema, pulmonary crackles, or chest radiography primarily assess extracellular fluid and do not accurately reflect intravascular volume status or venous congestion. This review explores the rationale, mechanism, and evidence behind more recent methods used to assess volume status, namely, lung ultrasound, inferior vena cava (IVC) ultrasound, venous excess ultrasound score, and basic and advanced cardiac echocardiographic techniques. These methods offer a more accurate and objective assessment of fluid status, providing real-time, non-invasive measures of intravascular volume and venous congestion. The methods we discuss are primarily used in inpatient settings, but, given the increased pervasiveness of ultrasound technology, some could soon expand to the outpatient setting.

## 1. Introduction 

Fluid or volume overload, a prevalent complication in patients with renal disease and hypertension, significantly impacts patient morbidity and mortality [1,2]. One of the clinical daily challenges that clinicians face is to identify fluid overload early enough in the course of the disease to prevent adverse outcomes and to guide and potentially reduce the intensity of the diuresis. This review will examine the impact of volume overload and its diagnosis by conventional methods and will discuss the rationale and evidence behind more recent methods used to assess volume status, namely, lung ultrasound, inferior vena cava (IVC) ultrasound, venous excess ultrasound score, and basic and advanced cardiac echocardiographic techniques.

## 2. Impact and Consequences of Volume Overload 

Volume overload is defined as the pathologic accumulation in the body of sodium and water. There is no agreed-upon threshold above which a patient can be deemed to be in volume overload, but some authors use a value of a 5% increase in body weight accumulating over a short period of time [3,4].

The development and maintenance of a state of volume overload occurs when one or more of the following factors are present: excess salt and fluid, cardiac dysfunction, renal failure, and neurohormonal activation [5]. This has been reviewed in detail by others [6]. Some of the hallmarks of volume overload are elevated cardiac filling pressure and central venous pressure (CVP). Since the venous system resistance is low, the elevated CVP gets transmitted to the inferior vena cava, and the hepatic and renal veins. Renal perfusion pressure (arterial minus venous pressure) drops as the venous side becomes more congested. As this occurs, renal function worsens and neurohormonal activation and sodium and fluid retention ensues, further worsening the volume overload state [5,6,7].

Over time, the excess fluid leaves the vessels and accumulates in the interstitial space. This leads to numerous adverse organ effects and a state of clinical congestion. In the lung, there will be pulmonary edema, decreased lung compliance, and impaired air exchange [8], in the gastrointestinal tract impaired drug absorption and bacterial translocation [7], in the brain delirium [9], and in the kidney AKI [6,10].

Due to this clear impact on multiple organs, it is not surprising that volume overload is associated with worse short- and long-term outcomes in several clinical settings. In critically ill patients, there are lower survival rates [11], higher ICU length of stays, readmission rates, and time on the ventilator [12], and difficulty ambulating upon hospital discharge [13]. Patients with advanced chronic kidney disease have lower survival rates [14] and a more precipitous decline in renal function and higher renal replacement therapy rates with volume overload [15]. These higher mortality rates persist even after dialysis is initiated [1,16]. Fluid restriction in the perioperative period has also been shown to improve outcomes and reduce complications [17,18,19]. Finally, volume overload in patients with congestive heart failure is associated with an increased risk of all-cause death and heart failure readmission, with lower quality of life at discharge [20,21].

## 3. Diagnosis of Volume Overload

Given the importance of the identification of congestion and volume overload, it is imperative to diagnose it as early as possible to guide and adjust therapy. Traditionally, this has been carried out with careful history taking and physical examination. Unfortunately, the “classic” findings of clinical congestion (jugular venous pressure, pulmonary crackles, third heart sound (s3), and edema) are not only often absent (especially in the early stage) but can also be misleading. A meta-analysis of 22 studies on patients presenting to the emergency department (ED) with dyspnea found a low sensitivity for the diagnosis of congestion for the symptoms of orthopnea (50%) and paroxysmal nocturnal dyspnea (41%), and for the clinical signs of edema (51%), JVP (39%), and s3 (13%) [22]. A review of another set of studies that included critically ill patients, or patients presenting after myocardial infarction or electively for a procedure, found similar numbers in pool sensitivity (62%) and specificity (76%) [23]. In chronic dialysis patients, a study of 1106 patients estimated that almost half the patients with severe congestion had no lung crackles or peripheral edema on examination [24]. Moreover, clinical examination skills and abilities can vary greatly between clinicians due to differences in training and length of experience [25,26].

Due to these shortcomings, some have proposed enhancing our ability to diagnose volume overload with more objective measures such as laboratory tests. One of the most studied and widely used biomarkers is the brain-natriuretic peptide (BNP). BNP is released by the heart in response to volume overload and increased wall stress and induces vasodilation (by decreasing angiotensin II and norepinephrine), natriuresis and diuresis [27,28]. In a prospective randomized trial of 1586 patients presenting to the ED with dyspnea published more than 20 years ago, Maisel et al. demonstrated that a finding of non-elevated BNP essentially ruled out volume overload [29]. This result was confirmed in other studies [30,31]. This biomarker has some drawbacks, however, including false-positive values in patients with renal failure [32,33] and advanced age [34], and false-negative values in obese patients [35]. Furthermore, patients with preserved ejection fraction may not have elevated BNP despite a state of hemodynamic congestion and elevated cardiac filling pressures [36,37].

Chest radiographs have long been relied upon to identify patients with volume overload but unfortunately cannot be reliably used to rule out congestion since radiographic findings (interstitial edema, cardiomegaly, pleural effusion) frequently lag behind clinical signs. In a large study of 85,376 patients presenting to the ED, Collins et al. found an exceptionally high rate of false-negative results, with approximately 1 in 5 patients having no evidence of congestion on radiographs despite a diagnosis (later confirmed) of decompensated heart failure [38].

In summary, physical examination, blood biomarkers, and the use of chest imaging all inherently lack accuracy for the diagnosis of volume overload. Furthermore, chest radiographs and laboratory tests are not always readily available, and the results may be delayed. Ideally, a diagnostic modality that is best suited to diagnose volume overload should fulfill all the following criteria: high sensitivity and specificity, early diagnosis, low cost, ease of use, minimal side effects, and reproducible findings. The use of ultrasound fulfills to a great extent those criteria. 

## 4. Lung Ultrasound

Previously, it was thought that ultrasound (US) did not have a place in the diagnosis and management of parenchymal pulmonary diseases because sound waves do not travel in air. In fact, as recently as 2015 in Harrison’s Principles of Internal Medicine, the authors write: “US (…) is not useful for evaluation of the pulmonary parenchyma and cannot be used if there is any aerated lung between the US probe and the abnormality of interest” [39]. Over the past few years, however, US has been shown to be a rapid, accurate tool that can be used to diagnose and monitor (among others) parenchymal fluid and volume overload. 

**Figure 1:**
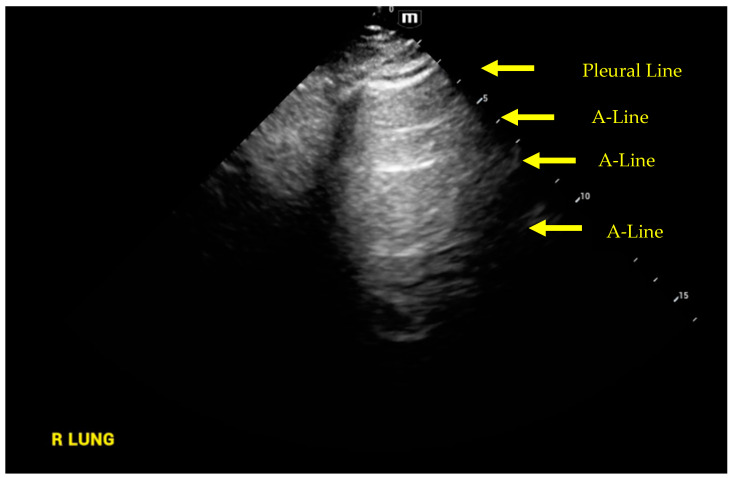
Normal lung ultrasound, A-line pattern.

After placing the patient in the recumbent position, the operator can use one of three protocols for image acquisitions: eight zones [40], twelve zones [41] and blue protocol [42]. As mentioned previously, the US beam is scattered by the air and therefore normal lung parenchyma will appear black on the US screen. The US will identify the pleural line (thickened white hyperechoic) and what have been called the A-lines (equidistant hyperechoic white lines) that are due to the interaction of the US probe with the pleural lining, creating a reverberation artifact. In Figure 1**,** we show a normal sector image of lung US with pleural line, A-lines, and dark space indicative of normal lung parenchyma. The presence of this pattern rules out interstitial edema.

When lung parenchyma fluid content increases, the US beam will produce a comet-tail artifact [43]. This artifact, also called B-lines, appears as a hyperechoic line that starts at and moves synchronously with the pleura and extends all the way to the bottom of the US image [40]. B-lines denote areas of expanded interlobular septa and increased lung water content. The presence of three or more B-lines on one image is an abnormal finding [40]. 

**Figure 2:**
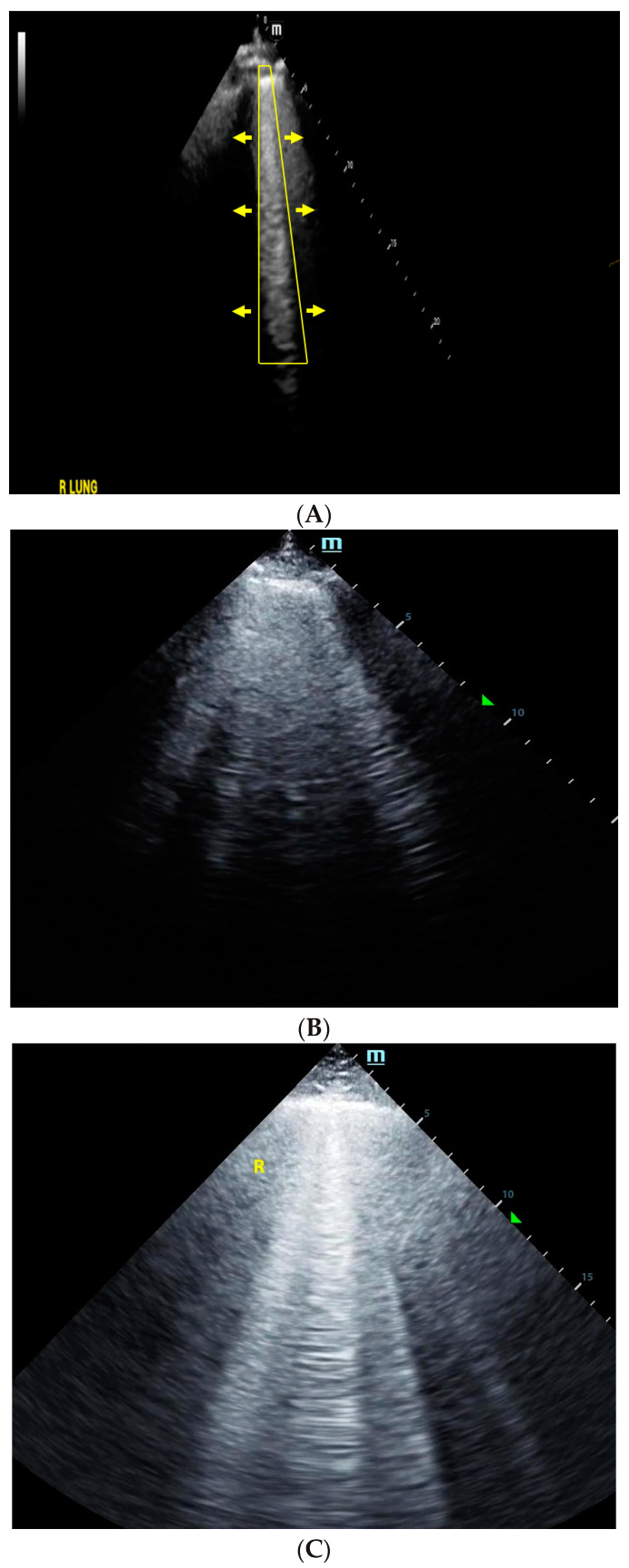
(**A**) Abnormal lung ultrasound, with B-line pattern reflecting possible interstitial edema. (**B**) Abnormal lung ultrasound, with B-line pattern indicating moderate interstitial edema. (**C**) Abnormal lung ultrasound, with B-line pattern indicating severe interstitial edema.

As pulmonary edema worsens due to volume overload, the number of B-lines increases and the B-lines coalesce into confluent B-patterns (Figure 2A–C) [40]. 

Multiple studies demonstrate a correlation between the number of B-lines on the US and the amount of lung water content [44,45,46,47]. As water content in the lung decreases, so does the number of B-lines [46]. There are several scoring systems that were developed using the number of B-lines and sectors examined but no consensus has been reached. Some experts recommend using a scoring system based on scanning 28 different points, whereas others opt for a more simplified approach [40,46,47,48]. 

Findings of B-lines does not necessarily indicate volume overload, however, as there are different conditions that can present with these findings. For example, if several B-lines are present in one or a few limited sectors, this could indicate a localized process such as a pneumonia, atelectasis, contusion, infarction, pleural disease, or neoplasia, whereas findings of diffuse B-lines may indicate pulmonary edema, interstitial pneumonia or pneumonitis, or diffused parenchymal lung disease like pulmonary fibrosis [40]. In the right clinical context, findings of B-lines have been shown to greatly improve the accuracy of diagnosis of congestion and volume overload. In a large study of 1005 patients, Pivetta et al. used US to evaluate patients who presented with acute dyspnea to the ED. The authors found that that US findings of B-lines improved the sensitivity and specificity of the diagnosis compared to clinical work-up alone (including chest radiograph and BNP). In total, 19% of patients were correctly reclassified using this modality [49]. In a randomized controlled study of 320 patients, the use of US was found to lead to the earlier diagnosis of congestion and the initiation of appropriate therapy in a substantially higher number of patients [50].

Lung ultrasound provides rapid noninvasive assessment of extravascular fluid across inpatient and outpatient settings in AKI/CKD. It allows dynamic monitoring of even small changes in pulmonary congestion that may be difficult to detect clinically to help titrate diuresis. The limitations of lung ultrasound include operator dependence and the fact that, without additional testing to evaluate left atrial pressure, it is difficult to determine cardiogenic versus non-cardiogenic pulmonary edema. However, it remains invaluable for extravascular fluid quantification in kidney disease through simple B-line scoring.

## 5. Inferior Vena Cava Ultrasound

IVC ultrasound utilizes measurements of IVC diameter and collapsibility with respiration to provide information on a patient’s central venous pressure [51,52]. While the patient is in the supine position, the operator measures the diameter of the IVC about 1 to 2 cm below the cavo-atrial junction. In non-ventilated patients, during the inspiratory phase there is a negative intrathoracic pressure that is elicited. This negative pressure results in an increase in filling of the right atrium and is transmitted to the IVC, which in turns collapses [51]. The integration of the size of the IVC and the percentage collapse using a sniff maneuver reasonably estimates right atrial pressure as follows: a size < 2.1 cm and >50% collapse is 3 mm Hg, a size < 2.1 cm and <50% collapse or a size > 2.1 cm and >50% collapse is 8 mm Hg, while a size > 2.1 cm and <50% collapse is 15 mm Hg or more [51,53,54]. Traditionally, the IVC is measured in the subcostal region; however, images cannot always be acquired in this position. A coronal trans-hepatic view has been offered as an alternative approach; unfortunately, measurements between these two views are not interchangeable [55]. To improve the accuracy, the use of an artificial intelligence program has shown a moderate correlation of IVC distensibility measurements between trans-hepatic and subcostal M-mode images in both the ventilated and spontaneously breathing patient [56,57].

Importantly, the most informative measurements of IVC diameter for assessing fluid responsiveness are carried out just caudal to the hepatic vein inflow in the IVC, compared to more cranial locations [58]. This allows for the calculation of the collapsibility index (cIVC), defined as (maximum diameter–minimum diameter)/maximum diameter in spontaneously breathing patients. It also enables calculation of the distensibility index (dIVC), defined as (maximum diameter–minimum diameter)/minimum diameter in mechanically ventilated patients to predict fluid responsiveness [59]. In spontaneously breathing patients, a cIVC of >40% indicates fluid responsiveness. Meanwhile, in mechanically ventilated patients, a dIVC cutoff of >18% predicts fluid responsiveness [60]. 

**Figure 3:**
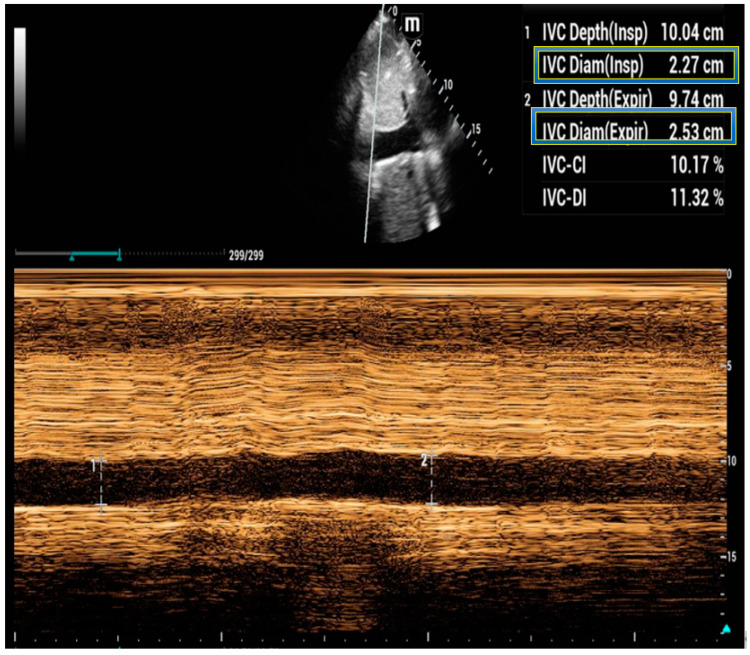
IVC ultrasound showing a diameter >2.1 cm with <50% change during the respiratory cycle.

Figure 3 shows a typical IVC image with a less than 50% change in diameter between inspiration and expiration.

The IVC assessment can help identify patients who are not in a volume overload state. In a prospective study of 136 patients with systolic heart failure presenting to the ED, an IVC diameter of less than 2.1 cm had a sensitivity of 90% and specificity of 73% to identify patients with compensated heart failure [61]. Furthermore, a dilated IVC has been shown to be associated with worse short- and long-term outcomes in both the ambulatory and hospital settings. In 693 patients with chronic heart failure, seen in a community clinic, IVC dilation was the strongest predictor of heart failure hospitalization and cardiovascular death at 3 years [62]. Similarly, in a retrospective review of a cohort of 335 patients from the ambulatory care setting, IVC dilation was associated with a higher likelihood (38% for every 0.5 cm increase) in heart failure admissions [63]. Finally, in 80 patients admitted to the hospital with acute decompensated heart failure, a dilated IVC on admission of 1.9 cm or more was associated with higher mortality rates at 90 and 180 days [64]. In that study, a value of 1.9 cm was also associated with hospital readmissions. 

IVC ultrasound integration with lung findings provides additional rapid volume status information and insights into venous congestion. Serial IVC assessments can help guide diuretic dosing, with a small decrease in IVC diameter or improved respiratory collapsibility suggesting diuresis response. The limitations of IVC ultrasound include variability in diameter measurements with different phases of respiration and a lack of standardized cut-offs that have been validated in CKD/AKI populations. In addition, young patients and athletes might have an IVC of greater than 2 cm despite a normal CVP [53,65,66].

## 6. Venous Excess Ultrasound Score

The venous excess ultrasound score (VExUS) integrates Doppler ultrasound findings into a semi-quantitative score reflecting the severity of systemic fluid overload and venous congestion [67].

**Figure 4:**
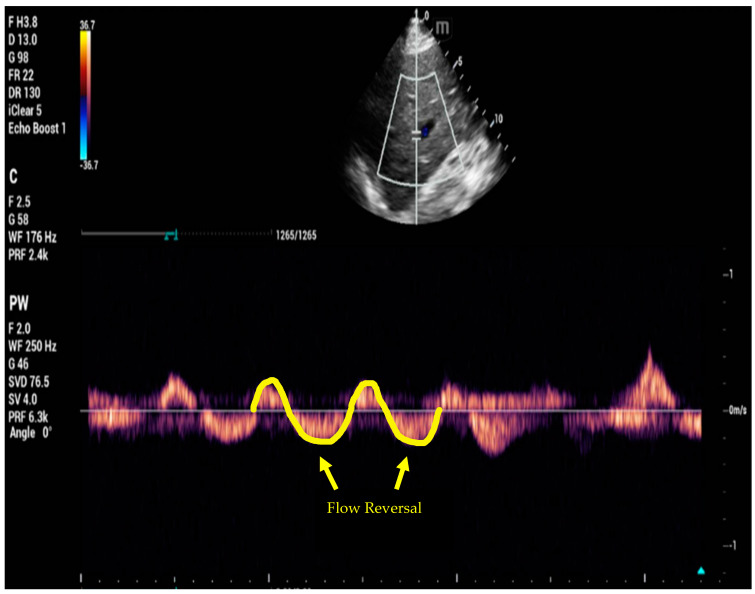
Doppler of hepatic vein showing flow reversal.

**Figure 5:**
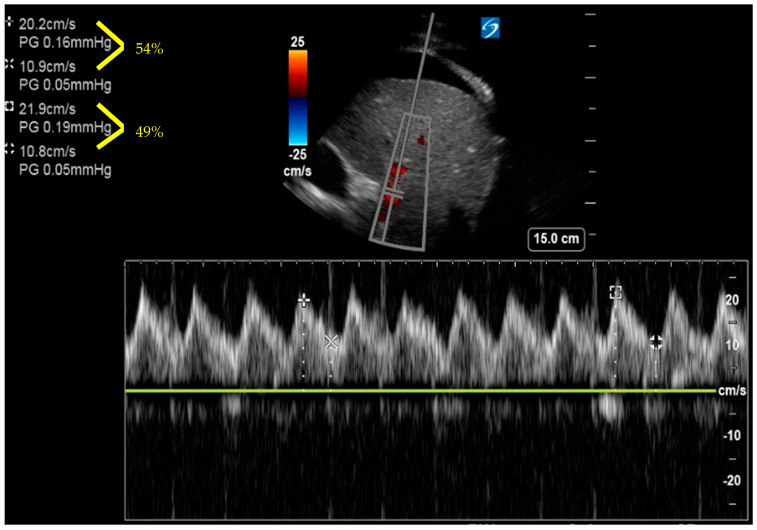
Doppler of portal vein showing >50% pulsatility index.

It incorporates four components: IVC, hepatic vein (Figure 4), portal vein (Figure 5), and renal vein evaluations. Each component is graded on a scale from 0 to 2 based on established cut-off values. The composite VExUS score ranges from 0 to 6, with higher scores suggesting worse fluid overload and more severe venous congestion. The operator has to be familiar with Doppler imaging and must use adequate imaging angle to avoid errors.

In the original study by Beaubien-Souligny, 145 patients were evaluated for the first 72 h after cardiac surgery using the VExUS grading system [67]. A severe VExUS grade on admission was associated with high likelihood of AKI (HR 2.82 after adjustment) and a favorable positive likelihood ratio (6.37) outperforming invasive central venous pressure monitoring. One caveat, however, is that this study was a post hoc analysis.

A prospective study of 51 ambulatory and inpatients examined the correlation between the VExUs score and right heart catheterization results. There was a significant association between a high VExUS grade and elevated right atrial pressure. Furthermore, compared to the IVC measurement, VExUS had an impressive AUC for the prediction of a RAP ≥ 12 mmHg (0.99, 95% CI 0.96–1) [68].

Other studies had more mitigated results. In a prospective study of 114 patients, some (hepatic Doppler flow) but not all (portal and infrarenal venous flow) components were associated with major adverse kidney events at 30 days [69]. Finally, in a prospective trial, 145 ICU patients were evaluated three times by VExUS during their stay (day 1, day 2, and last day). There was no significant association between the VExUS scores and AKI during the first week or 28-day mortality [70]. 

In summary, Doppler imaging may add some useful information regarding volume overload in selected patients. Integrating these findings into a larger clinical context may help facilitate the early diagnosis of AKI and in some patients potentially guide diuretic therapy [71,72]. It is important, however, to remember that Doppler abnormalities can be seen in patients without volume overload (such as the impact of stiff liver parenchyma in cirrhotic patients on portal flow pattern). One additional downside of this technique is that renal venous Doppler can be difficult to obtain. 

## 7. Basic and Advanced Echocardiography 

Limited cardiac echocardiography performed and interpreted at bedside by non-cardiologists (also called point-of-care ultrasonography or POCUS) has gained popularity in fields that require quick and effective answers to specific physiologic questions to improve diagnostic accuracy. It has become an integral part of the training in emergency medicine and critical care medicine. Using this imaging modality, the clinician can assess several parameters that are critical in volume overload states, namely, the left ventricle systolic and diastolic function as well as cardiac filling pressures and the right ventricle size and function. 

The ejection fraction of the left ventricle can be visually assessed with quick “eye balling” (the “eye test”) and classified as normal, reduced, or severely reduced. Studies conducted with emergency physicians showed good agreement between their findings and those of advanced sonographers [73]. Other methods include fractional shortening, E-point septal separation, and the Simpson method [74]. 

**Figure 6:**
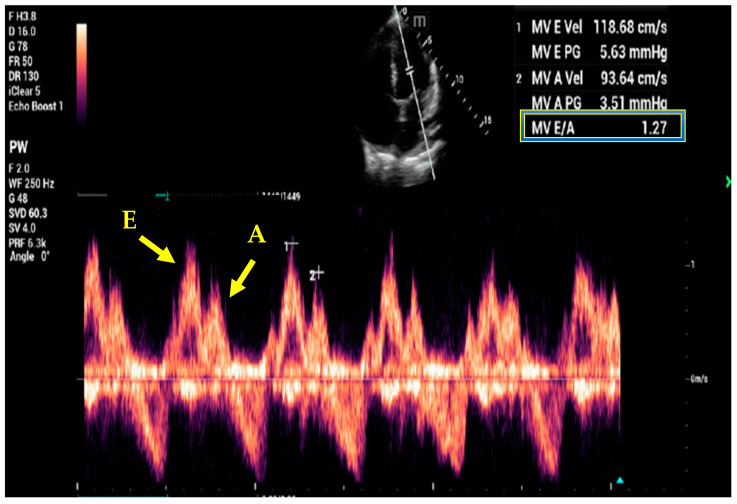
Doppler analysis showing mitral valve inflow including E (passive filling during early diastole) and A (active filling during late diastole as the LA contracts) waves with a normal E/A ratio.

**Figure 7:**
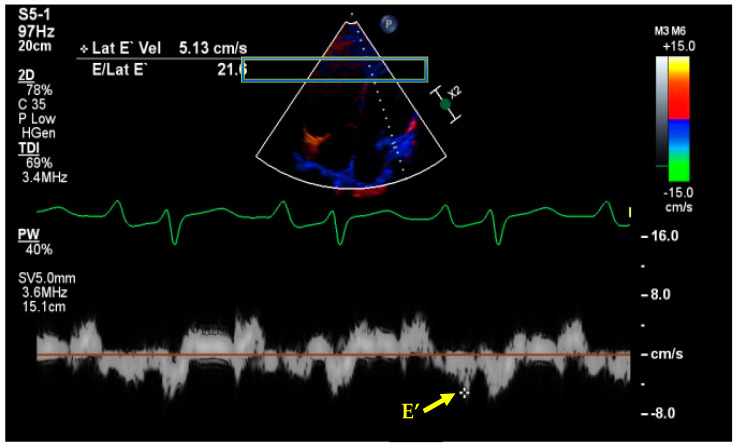
Tissue Doppler image (TDI) showing E’ and elevated E/E’ ratio implying elevated filling pressures. E wave calculated from Doppler analysis over MV inflow (not shown).

Doppler analysis of the mitral inflow can help identify patients with diastolic dysfunction and impairment. When performing this study, patients in sinus rhythm have E (early diastolic filling phase) and A (late diastolic filling due to atrial contraction) waves (Figure 6). The additional measurement of tissue Doppler imaging (TDI) can identify patients with elevated cardiac filling pressures (Figure 7). An elevated E/E’ (obtained using TDI) is indicative of elevated left filling pressures [75,76,77]. A simplified definition of diastolic dysfunction has been recently described requiring only measurement of e’ and E/e’ [78]. This is the most used approach in the critical care setting as even the non-proficient sonographer can use it [79]. The assessment of diastolic dysfunction can be carried out by assessing e’ and E/e’ alone, and right ventricle size and abnormal movement of the septum can indicate pressure or volume overload [80]. Similarly to the mitral flow E/E’ calculation, the tricuspid E/E’ can estimate right atrial pressure [81]. In one interesting study of outpatients with heart failure, the management of patients using echo- (E/E’) and BNP-guided management was found to be associated with lower mortality, decreased decline in renal function, and lower need for diuretics compared to clinically guided management [82].

Basic and advanced echocardiography performed by clinicians at the bedside requires training in both image acquisition (and optimization) as well as interpretation. Several views of the heart (parasternal, apical four chambers, apical five chambers, subcostal) need to be obtained to get an accurate assessment. This information, however, is invaluable as it provides real-time data that can be incorporated into the overall management of the patient. 

## 8. Conclusions

The accurate quantification of volume overload remains challenging but critical. There are several limitations to physical examination, chest radiographs, and laboratory tests. Through the direct visualization of fluid status, point-of-care ultrasound shows immense promise to improve volume assessment and management. While a significant amount of the research has focused on hospitalized patients, point-of-care ultrasound has growing potential for fluid status assessment in the outpatient setting. Portable handheld ultrasound devices are becoming increasingly available and affordable and provide clinicians with rapid non-invasive tools to quantify volume overload and guide management across different settings. However, important challenges remain, including substantial operator dependence, required training, inter-observer variability, and a lack of standardization of protocols and interpretive criteria that may affect the reproducibility of these emerging techniques. While additional research is needed to validate and standardize approaches, point-of-care ultrasound holds significant promise as an accessible, non-invasive tool to assess fluid status and guide clinical decision making.

## Data Availability

Not applicable.
